# Novel Multiarm Polyethylene glycol-Dihydroartemisinin Conjugates Enhancing Therapeutic Efficacy in Non-Small-Cell Lung Cancer

**DOI:** 10.1038/srep05871

**Published:** 2014-07-29

**Authors:** Lin Dai, Luying Wang, Lihong Deng, Jing Liu, Jiandu Lei, Dan Li, Jing He

**Affiliations:** 1 MOE Key Laboratory of Wooden Material Science and Application, Beijing Forestry University, Beijing 100083, P. R. China

## Abstract

The clinical application of dihydroartemisinin (DHA) has been hampered due to its poor water-solubility. To overcome this hurdle, we devised a novel polymer-drug conjugate, multiarm polyethylene glycol-dihydroartemisinin (PEG-DHA), made by linking DHA with multiarm polyethylene glycol. Herein, we investigated PEG-DHA on chemical structure, hydrolysis, solubility, hemolysis, cell cytotoxicity *in vitro*, and efficacy *in vivo*. The PEG-DHA conjugates have showed moderate drug loadings (2.82 ~ 8.14 wt%), significantly good water-solubilities (82- ~ 163-fold of DHA), excellent *in vitro* anticancer activities (at concentrations ≥8 μg/ml, showed only 15–20% cell viability) with potency similar to that of native DHA, and long blood circulation half-time (5.75- ~ 16.75-fold of DHA). Subsequent tumor xenograft assays demonstrated a superior therapeutic effect of PEG-DHA on inhibition of tumor growth compared with native DHA. The novel PEG-DHA conjugates can not only improve the solubility and efficacy of DHA but also show the potential of scale-up production and clinical application.

Lung cancer is the most common cause of cancer-related morbidity and mortality, resulting in >1.1 million deaths per year worldwide^1^,^2^. Non–small-cell lung cancer (NSCLC) represents more than 80% of lung cancer diagnoses, and has an overall 5-year survival rate of approximately 16% which decreases precipitously among patients diagnosed with latestage disease^3^,^4^. In this situation, chemotherapy is still a mainstay treatment.

Dihydroartemisinin (DHA) is a derivative of artemisinin, the active principle of the Chinese medicinal herb Artemisia annua^5^,^6^. Recommended by the WHO, the drug has been used for treating more than 2 million cases of malarial infection, mainly in Africa and Asia^7^. Artemisinin and its analogs are considered as safe drugs with no obvious adverse reactions or noticeable side effects^8^. Recently, it has been found that DHA has antiproliferative effects on various tumor cell lines including cancers of the breast, colon, pancreas, liver, lung, ovary and prostate^9^,^10^,^11^,^12^. Moreover, some research has also shown that DHA can significantly reduce the level of c-MYC protein, which leads to cell cycle arrest and apoptosis in tumors cells^13^. Although DHA has demonstrated an anticancer activity, it still has some drawbacks, such as low bioavailability, caused by its poor solubility in solution and blood, an initial burst release effect, and high peak plasma concentration from its rapid metabolism^14^,^15^,^16^. Therefore, increasing the solubility of DHA is necessary to achieve the above-mentioned therapeutic or anticancer effects^17^.

Many attempts to increase the solubility of DHA have focused on stable substitutions at hydroxyl radical^18^, but the substitutions have no effect on the half-time *in vivo*. PEGylation with high solubility in water, minimal side effects, and well defined molar mass may provide a solution to these problems. An alternative strategy is to modify the lactone moiety reversibly through macromolecules in order to create a water-soluble “prodrug” capable of releasing active-lactone DHA via hydrolysis processes. “Simple” polyethylene glycol (PEG) carrier can substantially enhance the properties of the drugs. The most obvious effect of PEGylation in this system is to increase the solubility and sustained release of macromolecular drugs, which in turn increases the cellular drug availability, decreases toxicity and enhances specific activity^19^,^20^,^21^,^22^. However, a traditional linear PEG has only two functional groups available for the conjugating one or two drug molecules though covalent bonds, so the linear structure of PEG limits the loading capacity for small molecule drugs^23^,^24^. In addition, some investigators claim that the PEG with a comparatively low molecular weight may have toxic effects, and a large molecule impede the release of drugs with small molecular weights, so that drugs do not reach therapeutic concentrations at the target sites^25^,^26^.

To overcome these potential shortcomings, in this study, we proposed to synthesize a novel prodrug system for insoluble anticancer drug DHA by using multiarm PEG (4 or 8 arms) with more functional groups and the appropriate molecular weight (20 KDa or 40 KDa, Figure 1a), in which the 10-hydroxy group of DHA was bound to carboxylic group of PEG using 1-ethyl-3-(3-dimethylaminopropyl)-carbodiimide hydrochloride as coupling reagent and 4-dimethylaminopyridine as the organic base (Figure 1b). The obtained multiarm polyethylene glycol-dihydroartemisinin (PEG-DHA) was characterized to show the physicochemical properties and *in vitro* release profiles. The cytotoxicity *in vitro* was investigated by human lung cancer cells (A549) and lewis lung carcinoma cells (LLC), and also the pharmaceutical effects of these prodrugs *in vivo* were assessed on the C57BL/6 mouse model.

## Results

### Synthesis of 4armPEG_40K_-DHA, 8armPEG_40K_-DHA and 8armPEG_20K_-DHA conjugates

**Figure 1:**
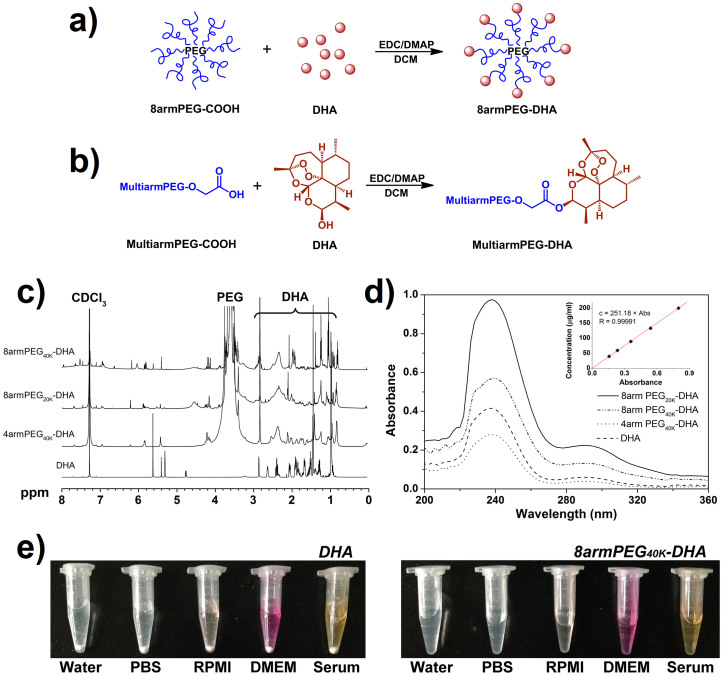
Design, synthesis and characterization of multiarmPEG-DHA conjugate. (a) Experimental design of 8armPEG-DHA conjugate. (b) Synthesis of multiarmPEG-DHA conjugate. (c) ^1^H-NMR spectra of DHA and multiarmPEG-DHA conjugates. They were solubilized in CDCl_3_ for ^1^H-NMR analysis (600 MHz). Peaks of PEG and DHA were indicated, respectively. (d) Absorbance spectrum of DHA and multiarmPEG-DHA conjugates in UV-Vis buffer. (Inset) Linear regression fit of DHA standards to calculate the concentration for DHA (n = 3 tests, 4 scans per test, R = 0.99991). (e) DHA and 8armPEG_40K_-DHA in different solutions recorded after centrifugation at 3,000 rpm for 5 min.

Figure 1c showed the ^1^H-NMR (CDCl_3_) spectra of DHA, 4armPEG_40K_-DHA, 8armPEG_40K_-DHA and 8armPEG_20K_-DHA, where the signals at 0.8 ~ 2.9 attributed to the most characteristic peaks of protons of DHA^27^, 3.50 ~ 3.85 (4nH, –(CH_2_CH_2_O)_n_–) and 4.20 (2H, –CH_2_OC(O)O–) attributed to the methylene protons of PEG^28^,^29^. The multiplet around 4.78 (1H, CH) of DHA moving to 5.82 (1H, CH) in PEG-DHA spectra indicated successful synthesis of ester bonds between PEG and DHA. The internal standard method by UV-Vis was used to further confirm that DHA was conjugated to PEG.

### Drug-to-carrier ratios

The absorbance spectra of DHA and PEG-DHA were measured by UV-Vis spectrophotometer (Figure 1d). From the standard plot of concentration vs absorbance, the coefficient (*Δ*) for DHA was calculated to be 251.18 μg/ml. The 4armPEG_40K_-DHA, 8armPEG_20K_-DHA, and 8armPEG_40K_-DHA solutions were diluted into 3.5 mg/ml, 2.0 mg/ml, and 6.0 mg/ml, respectively; and then the UV absorbance of them at 238 nm was determined after pretreating solutions^30^. Using the absorbance value, and employing the coefficient *Δ* obtained from above, the concentration of DHA in the sample was determined. Thus, dividing the DHA concentration by the PEG-DHA provided the percentage of DHA in the conjugates (Table 1). The mean mass ration of drug-to-carrier for 4armPEG_40K_-DHA, 8armPEG_40K_-DHA, and 8armPEG_20K_-DHA was 2.82 ± 0.16%, 4.70 ± 0.13%, and 8.14 ± 0.28%; and the molar ration of them was 3.29 ± 0.23, 6.94 ± 0.18, and 6.24 ± 0.20, respectively. The mass/molar ration indicated that approximately 1 or 2 functional groups of multiarm PEG remained unconjugated.

### Solubility study

Solubility studies were conducted to investigate the solubility of PEG-DHA conjugates in various solvents (Figure 1e). As predicted, PEGylation has substantially increased the solubility of DHA, which is an insoluble molecule by itself (Table 1). DHA is almost insoluble in water (<0.1 mg/ml) which was referred by Pharmacopoeia of People's Republic of China^31^. All the PEG-DHA conjugates have 199.8 to 289.0 mg/ml solubility in water, which is equivalent to as high as 16.26 mg/ml of DHA. This highly increased solubility (82 ~ 163 times increase) makes it possible to systemically evaluate the therapeutic efficacy of DHA *in vivo*.

### Stability study

**Figure 2:**
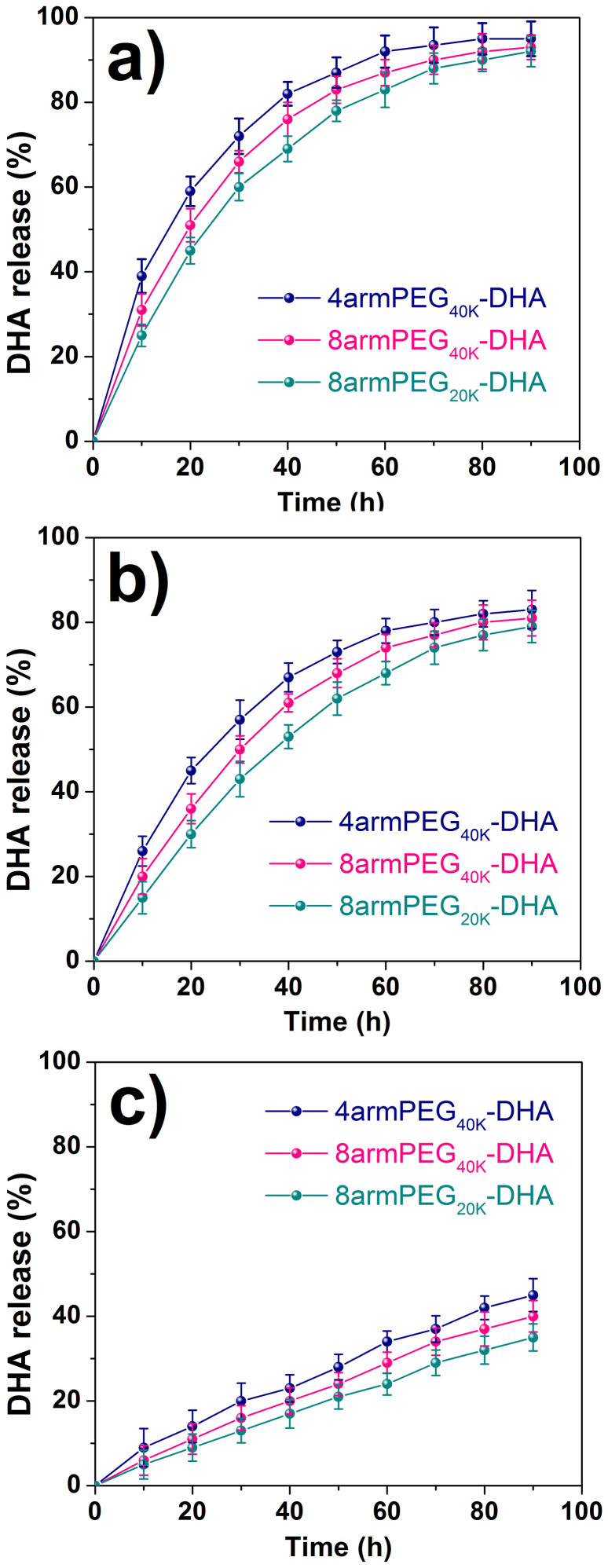
Stability of PEG-DHA conjugates in water at 37°C. Experiments were done at pH 8.1 (a), at pH 7.4 (b), and at pH 6.1 (c) for 4armPEG_40K_-DHA, 8armPEG_40K_-DHA, and 8armPEG_20K_-DHA. The presence of native DHA was monitored as a function of time. Data was acquired by UV-Vis analysis (n = 3, error bars represent standard deviation).

As the PEG-DHA conjugates were synthesized through covalent bonds between the carboxylic groups of PEG and hydroxide radicals of DHA, we expected the hydrolytic cleavage of ester bonds to occur before the drug can exert a significant cytotoxic effect^32^. Hydrolysis studies demonstrated that native DHA is released from PEG-DHA conjugates in PBS and the hydrolysis rate is strongly dependent on pH. PEG-DHA conjugates were placed in aqueous solutions that simulated biological fluids to measure the rates of hydrolysis by UV-Vis analysis. The stabilities of PEG-DHA conjugates were determined in buffered solutions at pH 8.1 (Figure 2a), pH 7.4 (Figure 2b), and pH 6.1 (Figure 2c) at 37°C to simulate the blood environment^33^,^34^. In the preclinical studies, the release of DHA could be due to several mechanisms as previously proposed^35^. The resultant hydrolysis data is shown in Figure 2 and the half-lives for these curves are given in Table 1. All conjugates were much more stable at pH 6.1 than at pH 7.4 or 8.1, and the stability trend was the same as the trend of hydrolysis rates for the three conjugates (8armPEG_20K_-DHA < 8armPEG_40K_-DHA < 4armPEG_40K_-DHA). The hydrolysis half-lives of the conjugates were increased 3-times and 5-times at pH 6.1 over pH 7.4 and pH 8.1 values, respectively.

### Hemolysis study

**Figure 3:**
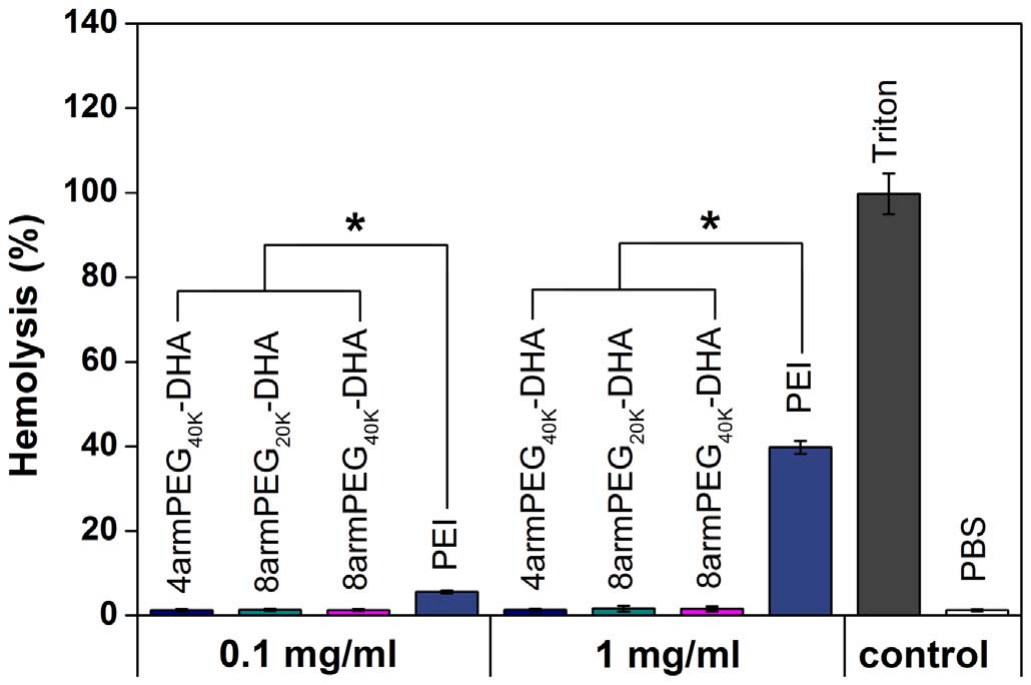
*In vitro* hemolysis assay of PEG-DHA conjugates compared to PEI_25K_ and Triton X-100 measured at 541 nm (n = 3, error bars represent standard deviation).

The detrimental interactions between conjugates and blood constituents such as the red blood cells (RBCs) must be avoided when injecting the conjugates into the blood circulation as a carrier for drug delivery^35^. Erythrocytes were incubated with two concentrations of polymer, 1 mg/ml and 0.1 mg/ml, for 1 h at 37°C. Hemolysis was evaluated by measuring the amount of hemoglobin released in the supernatant at 541 nm (Figure 3), and Triton X-100 was used to induce full hemoglobin release. Each kind of PEG-DHA (4armPEG_40K_-DHA, 8armPEG_20K_-DHA and 8armPEG_40K_-DHA) at 0.1 mg/ml and 1 mg/ml showed a comparable hemoglobin release to blank values (<2%), which was significantly lower than comparable concentrations of polyethylenimine (PEI_25K_), a cationic polymer known to have a significant hemolytic effect^26^. Moreover, DHA is not cytotoxic to the RBCs in a previous study^36^, suggesting the excellent safety of PEG-DHA conjugates.

### *In vitro* cytotoxicity

**Figure 4:**
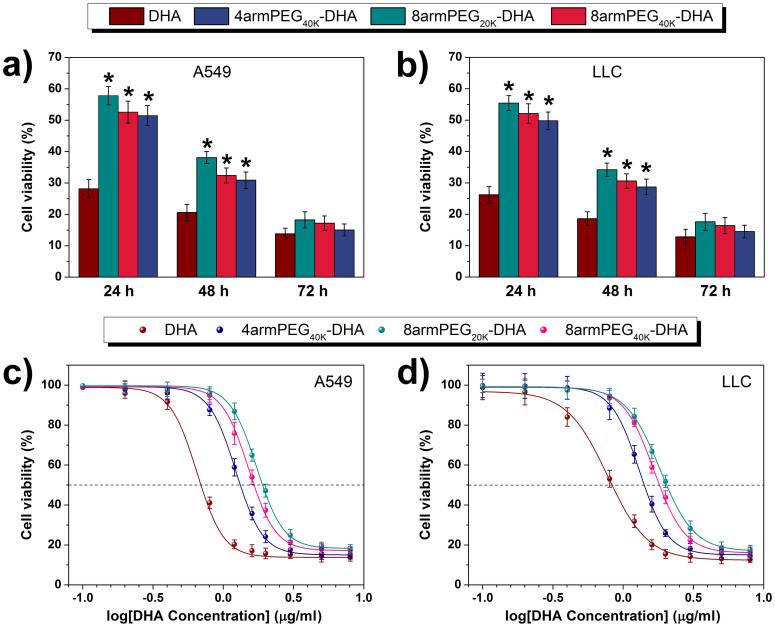
Cell viability of A549 (a) and LLC (b) cells treated with 8 μg/ml of DHA and PEG-DHA (equivalent to free BA) was measured by CCK-8 assay (n = 3, error bars represent standard deviation). CCK-8 assay of DHA and PEG-DHA conjugates with different concentration in A549 (c) and LLC (d) cell lines (n = 3, error bars represent standard deviation).

To ensure the effectiveness of the prodrug before their entry into human application, *in vitro* toxicity should be considered upfront^37^,^38^. To examine the cytotoxicity of DHA and PEG-DHA conjugates, a CCK-8 assay was conducted after incubating cells with different treated. The response of two cell lines (A549 and LLC) was tested *in vitro* by seeding the cells and exposing them to various concentrations of PEG-DHA conjugates, native DHA, or PEG. Cells were exposed to drug for 24 h, 48 h or 72 h. The analysis of vitro cytotoxicity measurements showed that DHA applied at 8 μg/ml induced cell death that was dependent upon the length of incubation, as shown in Figure 4a and 4b. At a dose of 8 μg/ml PEG-DHA conjugates, the viability was even reduced by ~85% and ~90% for LLC and A549 cells, respectively. PEG-DHA conjugates were equipotent to native DHA.

To compare the potency of conjugates, a drug concentration corresponding to 50% death of the cells (IC_50_) was estimated from survival curves in Figure 4c and 4d, obtained from replicate experiments. The IC_50_ of PEG-DHA conjugates were slightly greater than free drug (Table 2, column 1). All conjugates were sensitivity of cells with the trend in the IC_50_ values for the samples remaining the same (8armPEG_20K_-DHA > 8armPEG_40K_-DHA > 4armPEG_40K_-DHA > DHA). These values are virtually equivalent to that for the native drug, indicating that DHA is being released into the medium.

**Figure 5:**
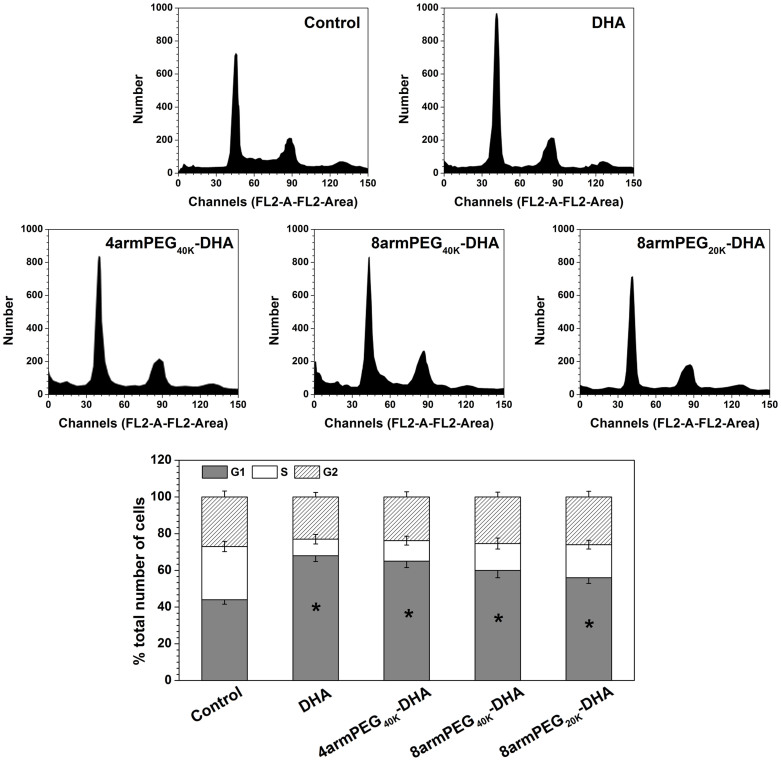
Inhibitory effect of DHA on cell cycle. LLC cell line exposed to IC_80_ of DHA for 24 h followed by cell cycle distribution assay. Values represent mean ± SD (n = 3).

Given the significant inhibitory effect of DHA and PEG-DHA conjugates on the viability of LLC cells, whether DHA could inhibit cell cycle progression was further examined. LLC cells was exposed to 20 μM of DHA and PEG-DHA conjugates for 24 h, and then collected for flow cytometry analysis. Significant changes in cell cycle were noted in LLC cell lines (Figure 5). As compared with the control group, the ratio of cells in G1 phase gradually increased. The ratio of cells in G1 phase are in the order DHA > 4armPEG_40K_-DHA > 8armPEG_40K_-DHA > 8armPEG_20K_-DHA, which is the same to the trend of the conjugates stability. The results indicate that DHA and PEG-DHA conjugates could all induce G1 phase arrest in LLC cells, and the PEG-DHA conjugates also have the similar bioactivity to DHA. In addition, our results indicate that G1 phase arrest is correlated with the extent of DHA release.

### The pharmacokinetics in mice

**Figure 6:**
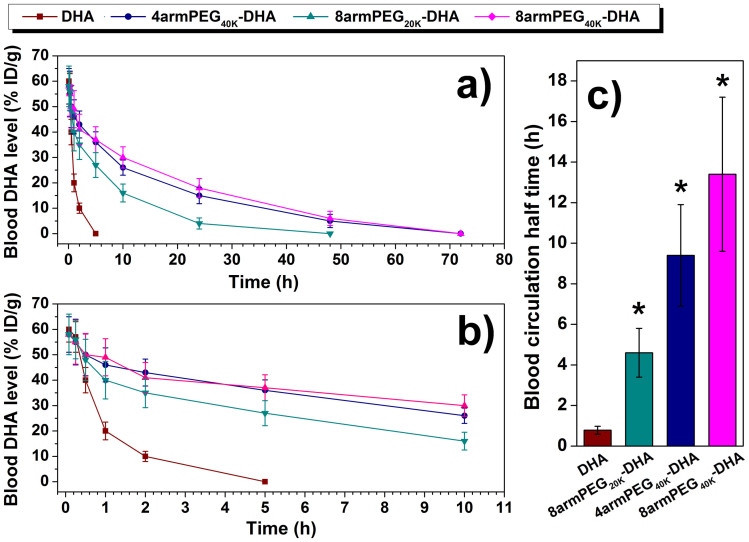
Blood circulation data in C57BL/6 mice. (a), (b) Blood circulation curves of PEG-DHA conjugates compared with free DHA. Error bars were based on six mice per group at each time point. (c) Blood circulation half-time of different PEG-DHA conjugates obtained.

Long blood circulation half-time of a drug carrier is desired to improve the bioavailability of the drug. The determined drug concentration after hydrolysis under basic condition was actually the total DHA in plasma, the combination of both parent form and conjugate form. The plasma clearance curves of free DHA and PEG-DHA conjugates in mice were shown in Figure 6a, b, and c. Disappearance of DHA from the blood circulation after intravenous administration of DHA injection was very rapid with the plasma concentration below 10% of injected dose per gram (% ID/g) at 2 h. 8armPEG_20K_-DHA removed a little slowly from the circulating system compared to free DHA, but the drugs were almost undetectable (4% ID/g) after 24 h. On the contrary, 4armPEG_40K_-DHA and 8armPEG_40K_-DHA exhibited a remarkable prolonged clearance with the drug levels of 15% and 18% ID/g at 24 h after administration. The blood circulation half-time of free DHA were 0.8 h. 8armPEG_20K_-DHA, 4armPEG_40K_-DHA, and 8armPEG_40K_-DHA could extend the blood circulation half-time of DHA from 0.8 h to 4.6 h, 9.4 h, and 13.4 h, respectively, which were far longer (5.75-, 11.75-, and 16.75-fold compared with DHA) than values of DHA. Additionally, the larger molecular weight of PEG-DHA conjugates was important for obtaining longer circulation.

### *In vivo* study

**Figure 7:**
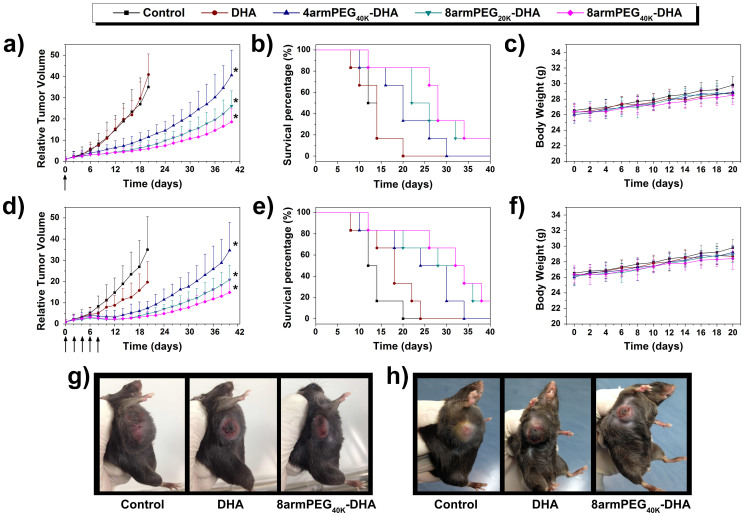
Antitumor efficacies of DHA and different DHA conjugates in the subcutaneous mouse model of LLC. Tumor volumes of mice during treatment with either a single (a) or multiple (q2d × 5) injections (d). Survival of mice in different treatment with either a single (b) or multiple (q2d × 5) injections (e). Body weight after treatment with either a single (c) or multiple (q2d × 5) injections (f). Tumor images of different groups after treatment with either a single (g) or multiple (q2d × 5) injections (h) at day 20 (n = 6, error bars represent standard deviation).

The results described above gave us great confidence to evaluate the anticancer effectiveness of DHA formulations in a mouse tumor xenograft model. LLC-tumor-bearing C57BL/6 female mice were injected intravenous with either DHA or PEG-DHA conjugates as a single dose of 50 mg/kg or multiple 10 mg/kg doses (every 2d, q2d × 5) schedule in xenograft models of lung tumor. On this cell line, the effectiveness of the samples are in the order 8armPEG_40K_-DHA > 8armPEG_20K_-DHA > 4armPEG_40K_-DHA > DHA. The treatment with a single-dose of 8armPEG_40K_-DHA resulted in 82.8% tumor growth inhibition (TGI) (on day 20) and 83.3% survival of animals (on day 24) (Figure 7a, 7b, and 7g, Table 3). However, a single dose of DHA given at the 50mg/kg had no effect on tumor growth. Multiple-dose treatment of 8armPEG_40K_-DHA caused 89.1% TGI, and by day 24, 83.3% of animals were survived. In contrast, multiple-dose DHA treatment resulted in 43.7% TGI (Figure 7d, 7e, and 7h, Table 4). No significant changes in body weight were noticed in all treatment groups compared to control group (Figure 7c and 7f).

## Discussion

DHA is shown to inhibit the growth of some human cancer cell lines with low toxicity, leading to a promising prospect in clinical application, but DHA suffers from low aqueous solubility and bioavailability^14^,^15^,^16^. So far many investigators have developed drug delivery systems for entrapping or conjugating DHA to a drug carrier in order to increase drug residence time at the site of pain and inflammation, decrease systemic exposure of immunosuppressants, and improve pharmacokinetics and preclinical efficacy.

In this study, we developed a new kind of prodrug based on multiarm PEG-DHA. Linear PEG is the most widely used for the simple synthetic steps and good water solubility, but the linear structure with only two functional groups limits the drug loading capability of PEG. While, multiarm PEG with a special molecular structure can be a better choice for highly efficient incorporation of drugs on the polymer scaffold. It has been widely demonstrated that polymers with a molecular weight >40 KDa display reduced renal clearance (enhanced pharmacokinetic), and are able to migrate through open malignant neovasculature and accumulate in tumors via the enhanced permeability and retention (EPR) effect^39^. Here the multiarm PEG was selected with very appropriate molecular weight (~40 KDa) and more functional groups (four or eight). PEG-DHA conjugates were synthesized via an esterification reaction between the carboxy groups of multiarm PEG-COOH and the hydroxyl group of DHA by a one-step method (Figure 1a and 1b). The PEG-DHA we generated is a more soluble DHA variant, owing to the hydrophilic ether bonds of PEG and enabled facile conjugation of DHA to a carrier compounds with an ester bond susceptible to hydrolysis^40^. *In vitro* release study showed significantly slower release kinetics for the conjugates (Figure 2). Since the ester bonds between the PEG and DHA hydrolyze and release the intact DHA under basic conditions. As expected, when treated with buffers, the releasing rate of DHA increased with increasing the pH from 6.1 to 8.1 in phosphate buffer at 37°C (Figure 2, Table 1). As a result, all the PEG-DHA conjugates show a potential to break the ester bonds, which further suggests that the anticancer activity of DHA can be regenerated during the incubation with tumor cells.

*In vitro*, PEG-DHA conjugates showed potent effects on A549 and LLC cell lines; however, there is a little difference in sensitivity to different cell lines. This could be due to differences in either the rate of releasing, intracellular delivery of native DHA, or resistance of cells to native DHA. PEG-DHA conjugates were comparable to native DHA in antitumor activity with IC_50_ in the low concentration range (Figure 4c and 4d). The IC_50_ of the conjugates correlated with the hydrolytic stabilities of the compounds in PBS, suggesting that a little more conjugates were needed to kill an equivalent fraction of cells and more released DHA from the PEG-DHA conjugates may lead to more cytotoxicity. *In vitro* experiments the tumor cell culture does not capture the advantages of PEGylating DHA compared to native DHA, such as improved pharmacokinetics, and hence may underestimate the efficacy of PEG-DHA conjugates.

It was confirmed *in vivo*, as expected, that all PEG-DHA conjugates demonstrated long blood circulation half-time, excellent anticancer activities and showed improved therapeutic efficacy of PEG-DHA over DHA in a mouse tumor xenograft model. There was almost no body weight loss in all the cured mice. They all were superior to DHA since the treatment with DHA resulted in either little TGI (single dose) or partial TGI sustained for a short period of time (multiple-dose regimen). The much enhanced antitumor efficacy of the PEG-DHA conjugates with appropriate molecular weights could be partially attributed to the high bioavailability due to the excellent water solubility, the prolonged tumor residence time, and the passive tumor targeting effect due to the EPR effect^39^.

In conclusion, a conjugate of one multiarm PEG molecule with several DHA molecules can effectively solubilize DHA. DHA conjugated in this prodrug system shows a significantly slow DHA release kinetics. PEG-DHA conjugate well retains the biological activity of DHA, and the 8armPEG_40K_-DHA and 4armPEG_40K_-DHA are both more active in cytotoxicity than 8armPEG_20K_-DHA *in vitro*. *In vivo* treatment using PEG-DHA conjugates was significantly more effective than DHA in the LLC xenograft model. Due to the excellent anticancer efficacy as well as the straightforward chemistry with little or no inherent impurities generated during the transformations, high drug loading capability and appropriate molecular weight, the 8armPEG_40K_-DHA was selected as the lead candidate for further preclinical development.

## Methods

### Synthesis of multiarm PEG-DHA conjugates

Four arm Poly(ethylene glycol) carboxylic acid (4armPEG-COOH, Mw = 40 KDa), eight arm Poly(ethylene glycol) carboxylic acid (8armPEG-COOH, Mw = 20 KDa, 40 KDa), were received from JenKem Technology Co., Ltd. (Beijing, China), and is an FDA and EU foodgrade material. Dihydroartemisinin (DHA) was obtained from Beijing Norzer Pharmaceutical Co., Ltd. 40 KDa 8armPEG-COOH (10.0 g, 0.25 mmol) and DHA (1.42 g, 5.0 mmol) were dissolved with 250 ml of dichloromethane (DCM). The solution was cooled to 0°C and 1-ethyl-3-(3-dimethylaminopropyl)-carbodiimide hydrochloride (EDC HCl) (0.58 g, 3.0 mmol) and 4-dimethylaminopyridine (DMAP) (0.61 g, 5.0 mmol). The mixture was stirred at 0°C for 1 h and at room temperature overnight. The solvent was evaporated under vacuum. The residue was dissolved in 100 ml of tetrahydrofuran (THF), and the crude product was precipitated with ethyl ether (500 ml). After filtration, the resulting solids were recrystallized with a mixture of N,N-dimethylformamide/isopropyl alcohol (DMF/IPA) (120 ml/480 ml). Then, the solids were filtered, washed with ethyl ether (2 × 500 ml), and dried under vacuum at 40°C to give 8armPEG_40K_-DHA. 4armPEG_40K_-DHA and 8armPEG_20K_-DHA were similarly synthesized and purified as that for 8armPEG_40K_-DHA (Table 1). Samples were dissolved in deuterated chloroform (CDCl_3_) for analysis by ^1^H-NMR (Bruker DRX-600 Avance III spectrometer).

### Drug-to-carrier ratio characterization

A mass and molar drug/carrier ratio for the PEG-DHA conjugate was determined as described here. DHA was detected by UV-Vis spectrophotometer as reported earlier^30^. Briefly, DHA were dissolved in 75 ml 60% (wt) of ethanol water solution, filter. 5 ml of the filter was mixed with 23 ml 2% (wt) of NaOH water solution and water bath heating at 60°C for 30 min. Then The UV absorbance of the derivative of DHA was determined at 238 nm for five different concentrations ranging from 40 to 200 μg/ml. The pretreatment of PEG-DHA conjugates was the same as native DHA. A mass or molar of PEG-DHA (mConjug or nConjug) were diluted in a known concentration, and absorbance at 238 nm and the concentration of DHA in the sample was used to obtain the mass and molar of DHA (mDHA, nDHA). The mass and molar drug/carrier ratios were thus reported as mDHA/mConjug and nDHA/nConjug.

### Hydrolysis in buffers

Hydrolysis profiles were obtained in phosphate buffer at pH 6.1, 7.4 and 8.1. PEG-DHA conjugates (20 mg/ml) were diluted into phosphate buffered saline and maintained at 37°C throughout the course of the hydrolysis study. Aliquots were removed at different time points, high-speed centrifuged to get supernatant, pretreatment as described previously, and then analyzed by UV-Vis at 238 nm. The percentage was calculated on the basis of the absorbance of the sample at 0 to 90 h vs the initial absorbance. Each stability profile represents the average of two independent runs with the same sampling schedules. The standard deviation of each point is typically 2% or less.

### Solubility study

The solubility of the conjugate was investigated as reported earlier^41^. Briefly, excess amounts of PEG-DHA were added to the screw capped scintillation vials containing 10 ml of various solvents or purified water. The suspension was mixed at ambient temperature. An aliquot of the sample (5 ml) was taken at 24 and 48 h intervals. Each withdrawn sample was filtered using a 0.45 μm PTFE filter, and then analyzed by UV-Vis as described previously.

### Hemolysis assay

The hemolytic activity of polymer solutions were evaluated as described previously^42^,^43^. Briefly, fresh blood samples were collected through cardiac puncture from rats. Ten milliliters of blood was added with EDTA-Na_2_ immediately to prevent coagulation. The red blood cells (RBCs) were collected by centrifugation at 1500 rpm for 10 min at 4°C. After washing in ice-cold DPBS until the supernatant was clear, erythrocytes were diluted at a final concentration of 5 × 10^8^ cells/ml in ice-cold DPBS. 1 ml PEG-DHA conjugates solution (1 mg/ml and 0.1 mg/ml) was mixed with 1 ml erythrocyte suspension. DPBS and 1% Triton X-100 in DPBS were used as controls for 0% lysis and 100% lysis, respectively. Samples were incubated for 1 h at 37°C under constant shaking. After centrifugation at 1500 rpm for 10 min at 4°C, supernatant was analyzed for hemoglobin release at 541 nm using an infinite M200 microplate spectrophotometer (Tecan, Switzerland). Hemoglobin release was calculated as (OD_sample_ − OD_negative control_)/(OD_positive control_ − OD_negative control_) × 100%. Hemolysis was determined from three independent experiments.

### *In vitro* cell cytotoxicity

Human lung cancer cells (A549), murine Lewis lung carcinoma (LLC) cells were obtained from the Peking University Health Science Center (China) and were grown in the listed medium: A549 (RPMI 1640 with 10% FBS, 1% streptomycin-penicillin); LLC (DMEM with 10% FBS, 1% streptomycin-penicillin). All cell lines were maintained in an incubator supplied with 5% CO_2_/95% air humidified atmosphere at 37°C.

CCK-8 assay was used for cell viability of different samples^44^,^45^. Briefly, A549 cells were seeded at a density of 4 × 10^3^ cells/per well in 180 μl culture medium within a 96-well plate (Corning, USA) and incubated overnight. Then, the cells were treated with various samples (DHA, 4armPEG_40K_-DHA, 8armPEG_40K_-DHA, and 8armPEG_20K_-DHA) at 37°C in a humidified incubator with 5% CO_2_ for 24 h, 48 h, and 72 h, where the samples of the DHA and PEG-DHA conjugates were dissolved in dimethylsulfoxide (Merck, Darmstadt, Germany) and diluted into medium before assay and DHA dose ranged from 0.2 to 8 μg/ml. 20 ml of CCK-8 solution (Dojindo Laboratories, Kumamoto, Japan) was added to each well of the plate and incubated for another 1 h at 37°C. The absorbance at 450 nm was measured an infinite M200 microplate spectrophotometer. Percent viability was normalized to cell viability in the absence of the samples. The IC_50_ was calculated as polymer concentration which inhibits growth of 50% of cells relative to non-treated control cells according to Unger et al.^46^. IC_50_ was calculated using the Boltzmann sigmoidal function from Origin® 8.6 (OriginLab, Northampton, USA). Data are representative of three independent experiments.

Cell cycle was assessed by flow cytometry (Attune Acoustic Focusing Cytometer, Applied Biosystems, USA). 2.0 × 10^4^ LLC cells were seeded in 25 cm^2^ flasks with 10 ml of nutrient medium and kept at 37°C, 5% CO_2_, for 24 h. Next day, DHA and PEG-DHA conjugates at their IC_80_ values were added. Cells were treated during 24 h. The adherent cells and the supernatant were harvested, centrifuged (1,000 rpm, 3 min, 4°C) and the pellet was washed with DPBS. The cells were fixed with 1 ml ice cold 70% ethanol and the samples were kept at 4°C for 18 h prior to the analysis. Fixed cells were centrifuged and resuspended with 1 ml PBS. After further centrifugation, the cells were dissolved in 0.5 ml RNAse A (0.1 mg/ml) and incubated at 37°C for 30 min. 50 μl propidium iodide (PI) was added and the samples were kept in the dark during 30 min before the analysis. Each analysis was done recording 1.0 × 10^4^ events and the results were compared with untreated controls.

### Pharmacokinetic experiments in mice

C57BL/6 female mice (6–8 weeks) were purchased from the National Institute for the Control of Pharmaceutical and Biological Products. All animal experiments were performed in accordance with Guide for the Care and Use of Laboratory Animals, and approved by Experimental Animal Ethics Committee in Beijing. 24 tumor-free healthy C57BL/6 female mice were divided into four groups at random. Group 1 was treated with DHA injection, groups 2–4 with different PEG-DHA conjugates, respectively, via the tail vein. All groups were given a single dose of DHA (50 mg/kg) or PEG-DHA conjugates (equal to 50 mg/kg DHA). After intravenous administration, blood samples were collected at 0.083, 0.25, 0.5, 1, 2, 5, 10, 24, 48, 72 h from the orbital plexus and centrifuged immediately at 3,000 rpm for 10 min at 4°C. The plasma was frozen at −20°C until assay. To determine the level of total DHA in each plasma sample, 100 μl of plasma was mixed with 50 μl of 0.1 N NaOH for 15 min in water bath at 37°C, allowing the hydrolysis of the conjugate. After that, 0.1 N HCl (50 μl) was added, followed by 100 μl methanol. After vortexed for 2 min, the mixture was sonicated for 5 min and centrifuged at 5,000 rpm for 5 min. The clear supernatant was dried under nitrogen, reconstituted by 100 μl methanol before HPLC analysis^47^. The HPLC employs a VYDAC 214TP54 (C18, 5 μm, 4.6 × 250 mm) with a UV detector, using a gradient of 60% of acetonitrile in 0.05% TFA at a flow rate of 1 ml/min. Blood circulation data were plotted as the blood DHA or PEG-DHA conjugates levels with the unit of percentage of injected dose per gram (% ID/g) against time after injection.

### *In vivo* efficacy study

LLC cells (1 × 10^6^ cells/200 μl DMEM media) were subcutaneously inoculated into the shaved right lateral flank of C57BL/6 female mice 24 h post-irradiation. Treatments were started when tumor volume reached 100–150 mm^3^, and this day was designated as day 0. Non-tumor-bearing C57BL/6 female mice were injected intravenous with either DHA or PEG-DHA conjugates as a single dose of 50 mg/kg or multiple 10 mg/kg doses (every 2d, q2d × 5). It is important to note that the doses or concentrations of PEG-DHA conjugates in this study refer to DHA equivalents. For example, a dose of 50 mg/kg of 8armPEG_40K_-DHA means that the dose contains 50 mg/kg of DHA and 1063 mg/kg (21-fold higher) of whole conjugate, here the loading of DHA in the whole 8armPEG_40K_-DHA is 4.70%. The mice were randomly divided into the following groups: PBS, DHA, 4armPEG_40K_-DHA, 8armPEG_20K_-DHA, and 8armPEG_40K_-DHA were administrated via tail intravenous injection. The corresponding tumor volume data were collected by measuring tumor diameter with an electronic caliper every day.

Tumor volume was calculated using the formula: (L × W^2^)/2, where L is the longest and W is the shortest tumor diameter (millimeter). Relative tumor volume (RTV) was calculated at each measurement time point (where RTV was equal to the tumor volume at a given time point divided by the tumor volume prior to initial treatment). To monitor potential toxicity, we measured the weight of each mouse. For humane reasons, animals were killed and regarded as dead if the implanted tumor volume reached 5,000 mm^3^. To further evaluate the hematological toxicity of different PTX formulations, we collected 200 μl of blood of each mouse after final administration. Obtained blood was immediately evaluated by a blood cell analyzer (MEK-7222K, Japan).

### Statistical

All experiments in this study were performed at least three times, and the data were expressed as the mean ± standard deviation (SD). Statistical analyses were performed by analysis of variance (ANOVA). All statistical analyses were performed using a 95% confidence interval (p < 0.05).

## Figures and Tables

**Table 1:** Synthesis Detail and Properties of PEG-DHA Conjugates

					Drug/carrier ratio			Hydrolysis t_1/2_ (h)^b^		
compound	PEG (mmol)	DHA (mmol)	EDC (mmol)	DMAP (mmol)	mass (%)	molar	solubility (mg/ml)	pH 8.1	pH 7.4	pH 6.1
DHA	--	--	--		--		<0.1			
4armPEG_40K_-DHA	0.25	2.0	1.2	2.0	2.82 ± 0.16	3.29 ± 0.23	289.0 (8.15^a^)	15.5	24.1	104.5
8armPEG_40K_-DHA	0.25	5.0	3.0	5.0	4.70 ± 0.13	6.94 ± 0.18	241.3 (11.33)	19.8	30.0	117.2
8armPEG_20K_-DHA	0.25	10.0	6.0	10.0	8.14 ± 0.28	6.24 ± 0.20	199.8 (16.26)	23.7	37.6	150.0

^a^Equivalent to native DHA.

^b^Based on the release of DHA.

**Table 2:** *In Vitro* Cytotoxicity of PEG Conjugates (IC_50_, μg/ml)

compound	LLC	A549
DHA	0.83 ± 0.022	0.69 ± 0.026
4armPEG_40K_-DHA	1.41 ± 0.033	1.33 ± 0.032
8armPEG_40K_-DHA	1.82 ± 0.036	1.65 ± 0.035
8armPEG_20K_-DHA	2.04 ± 0.041	1.91 ± 0.038

**Table 3:** LLC Xenograft Model (50 mg/kg single dose): Efficacy Comparison

compound	mean TV ± SD (mm^3^)^a^	RTV^a^	TGI(%)^a^	Cures(%)^b^
control	4795 ± 1037	35.0 ± 15.6	0	0
DHA	4704 ± 1092	40.9 ± 9.5	−16.8	0
4armPEG_40K_-DHA	1254 ± 327	11.5 ± 3.0	67.1	33.3
8armPEG_20K_-DHA	907 ± 315	7.2 ± 2.5	79.4	50.0
8armPEG_40K_-DHA	648 ± 248	6.0 ± 2.3	82.8	83.3

^a^Mean tumor volume (TV), RTV, and % TGI data were taken at day 20. (By day 20, a significant percentage of control animals were euthanized due to excess tumor burden.)

^b^% cures were taken at day 24.

**Table 4:** LLC Xenograft Model (10 mg/kg q2d × 5): Efficacy Comparison

compound	mean TV±SD (mm^3^)^a^	RTV^a^	TGI(%)^a^	Cures(%)^b^
control	4795 ± 1037	35.0 ± 15.6	0	0
DHA	2069 ± 879	19.7 ± 9.7	43.7	16.7
4armPEG_40K_-DHA	862 ± 368	7.5 ± 3.2	78.6	66.7
8armPEG_20K_-DHA	588 ± 252	4.9 ± 2.1	86.0	66.7
8armPEG_40K_-DHA	410 ± 216	3.8 ± 2.0	89.1	83.3

^a^Mean tumor volume (TV), RTV, and % TGI data were taken at day 20. (By day 20, a significant percentage of control animals were euthanized due to excess tumor burden.)

^b^% cures were taken at day 24.
